# Following Darwin’s footsteps: Evaluating the impact of an activity designed for elementary school students to link historically important evolution key concepts on their understanding of natural selection

**DOI:** 10.1002/ece3.7849

**Published:** 2021-08-25

**Authors:** Xana Sá‐Pinto, Alexandre Pinto, Joana Ribeiro, Inês Sarmento, Patrícia Pessoa, Leonor R. Rodrigues, Lucía Vázquez‐Ben, Evangelia Mavrikaki, Joaquim Bernardino Lopes

**Affiliations:** ^1^ Research Centre Didactics and Technology in Education of Trainers Campus Universitário de Santiago University of Aveiro Aveiro Portugal; ^2^ Polytechnic Institute of Porto School of Education Porto Portugal; ^3^ University of Trás‐os‐Montes e Alto Douro Vila Real Portugal; ^4^ Centre for Ecology Evolution and Environmental Changes Faculty of Sciences University of Lisbon Lisboa Portugal; ^5^ Faculty of Educational Studies Department of Pedagogy and Didactics University of Coruña A Coruña Spain; ^6^ Faculty of Pedagogy and Primary Education National & Kapodistrian University of Athens Athens Greece

**Keywords:** conceptual fields, evolution education, history of science, teleological thinking

## Abstract

While several researchers have suggested that evolution should be explored from the initial years of schooling, little information is available on effective resources to enhance elementary school students’ level of understanding of evolution by natural selection (LUENS). For the present study, we designed, implemented, and evaluated an educational activity planned for fourth graders (9 to 10 years old) to explore concepts and conceptual fields that were historically important for the discovery of natural selection. Observation field notes and students’ productions were used to analyze how the students explored the proposed activity. Additionally, an evaluation framework consisting of a test, the evaluation criteria, and the scoring process was applied in two fourth‐grade classes (*N* = 44) to estimate elementary school students’ LUENS before and after engaging in the activity. Our results show that our activity allowed students to link the key concepts, resulting in a significant increase of their understanding of natural selection. They also reveal that additional activities and minor fine‐tuning of the present activity are required to further support students’ learning about the concept of differential reproduction.

## Graphical abstract

We hypothesize that exploring conceptual fields historically important for the discovery of natural selection could foster elementary school students’ understanding of evolution. We designed, implemented, and evaluated a problem‐based transdisciplinary learning activity to explore this hypothesis. Our results show that this approach effectively promoted elementary school students' understanding of evolution.

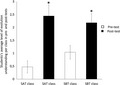

## 1 INTRODUCTION

Despite its fundamental importance to biology and many other research fields, several studies have shown that evolution is not understood as a valid scientific theory by many, with frequent and persistent misconceptions being shared by people across countries, ages, instructional levels, and developmental stages (Athanasiou & Mavrikaki, 2013; Bishop & Anderson, 1986; Miller et al., 2006; Nehm & Reilly, 2007; Prinou et al., 2011; Rutledge & Warden, 2000; Spiegel et al., 2012; To et al., 2017; Yasri & Mancy, 2016).

This is particularly worrying since understanding evolution is fundamental to understanding the surrounding world, making informed choices, and tackling personal and societal problems (National Research Council, 2012; Carroll et al., 2014). To overcome this problem, the NRC (2012) proposed evolution as one of the four core concepts in biology that should be explored since kindergarten and across students’ entire educational routes with increasing complexity. Few information is available on how evolution is incorporated in the curricula of the countries although a Framework to Assess the Coverage of biological Evolution by school curricula**—**FACE ‐ has been developed to analyze such question (Sá‐Pinto et al., 2021). However, despite the NRC’s (2012) recommendation, little information is yet available regarding effective strategies to teach evolution at such young ages or what students in elementary schools can learn about evolution, their knowledge, and misconceptions about this topic, although we know that, for older students, these vary a lot between different countries (Kuschmierz et al., 2020). Moreover, few studies have analyzed elementary school students’ understanding of evolution and even fewer explored their understanding of natural selection (Nadelson et al., 2009; Campos & Sá‐Pinto, 2013; Kelemen et al., 2014; Shtulman et al., 2016; Berti et al., 2017; Emmons et al., 2017; Sá‐Pinto, Pinto, et al., 2017; Sá‐Pinto, Cardia & Campos, 2017; Brown et al., 2020; Frejd et al., 2020).

Notably, discordant results were obtained in these studies regarding elementary school students’ ability to learn about natural selection after educational interventions. Campos and Sá‐Pinto (2013); Kelemen et al. (2014); Emmons et al. (2017); Brown et al. (2020); Frejd et al. (2020) reported that kindergarten and elementary school students (ages ranging from 5 to 10 years old) were able to understand and apply the principle of natural selection to explain and predict biological evolution following pedagogic interventions. However, in a study that tested a distinct pedagogical sequence, Berti et al. (2017) reported that only a minority of children (ages ranging from 7 to 9 years old) were able to learn about natural selection. Together, these results highlight the need for further studies analyzing elementary school students’ ability to learn about evolution by natural selection and about effective strategies to promote such learning.

Research in evolution education shows us that, unlike experts, novice students tend to be sensitive to the superficial features of a situation/problem (Nehm & Ridgway, 2011). For conceptually equivalent problems, students may provide different sets of normative and non‐normative ideas about evolution if these have distinct surface features (e.g., if the same problem is presented with animals evolving distinct traits or a plant is used instead) since these features activate distinct mental representations that will subsequently activate distinct concepts and problem‐solving schemas (reviewed in Nehm, 2018). Aligned with this view, Vergnaud (2009) argued that learning requires the development of conceptual fields, which he understands as a set of situations—that may be explored in different educational activities—and a set of linked concepts. Concepts and situations are tightly linked: A given situation can only be fully understood by applying and linking certain concepts, while the meaning of a concept can only be learnt by exploring a variety of distinct situations that highlight the set of a concept's invariants (i.e., objects, properties and relationships) that allow students to apply it to make sense of new situations and solve new problems (Vergnaud, 2009).

This emphasizes the need to have a set of good examples and educational activities that expose students to distinct situations involving evolution by natural selection that allow them to distinguish the concepts’ invariants from surface features and promote evolution understanding. This need contrasts with the scarcity of educational activities described to promote evolution understanding in elementary school students.

In his autobiography, Darwin described how facing distinct situations during the Beagle's voyage and after returning to England allowed him to develop his conceptual field related to evolution (Barlow, 1958). After returning to England, Darwin collected data and information from diverse sources about variation in wild and domestic animals and plants (Barlow, 1958). However, according to Darwin, the discovery of the process of natural selection only took place in October 1838, when he “*happened to read for amusement “Malthus* *on Population,” and being well prepared to appreciate the struggle for existence (...)* *it at once struck me that under these circumstances,* *favourable variations would tend to be preserved, and unfavourable* *ones to be destroyed. The result of this would be the formation of new species*” (Barlow, 1958, p.120). This sentence reveals the importance of contrasting the potential for the geometrical growth of natural populations with the constant or arithmetical growth of subsistence for Darwin to devise and operationalize the concept of natural selection. In support of this hypothesis, Wallace (Darwin & Wallace, 1858) used species’ potential for geometrical growth to depict the “*struggle for existence*” and to describe the evolutionary process that Darwin called natural selection. Both of these observations suggest that understanding the concept of natural selection may be facilitated by exploring Malthus’ principle.

While the specific situations Darwin and Wallace faced during their lives allowed them to discover evolution by natural selection, these are largely impossible to replicate in the classroom. Instead, we can design educational activities that require students to explore situations that address Malthus’ principle and to put in action concepts and conceptual fields that were important to the scientific discovery of natural selection. Therefore, our research question is: Will educational activities that require students to explore situations addressing Malthus’ principle and put in action historically important concepts and conceptual fields effectively promote students’ learning on evolution by natural selection? To answer this question, we aimed to i) design an elementary school level activity that uses a situation developed for students to explore Malthus’ principle and put in action key concepts and conceptual fields similar to those that were historically important for the scientific discovery of natural selection; ii) evaluate the impacts of the designed activity on students’ evolution understanding.

## 2 MATERIALS AND METHODS

To achieve our goals, we opted to use design research, as this methodological approach has been shown to be appropriate to develop and study educational practices and inform policies, especially when little is known about how to teach a given content (Barab & Squire, 2004; Van den Akker et al., 2006; Kelly, 2013). Design research consists of designing and implementing interventions aimed at solving a complex educational problem to either gain knowledge about the process of intervention design and development itself and/or validate new theories (Plomp, 2013).

Accordingly, two types of design research can be considered (i.e., development versus validation studies) and two principal outcomes can be obtained: the principles guiding the design of the activities for a given context and content and the interventions themselves. However, both orientations can be combined since they share the main features of the design research: both build on prior research and involve practitioners in the cyclical process of designing, evaluation and refining of the intervention (Plomp, 2013; Van den Akker et al., 2006).

Therefore, we present a study aimed at developing a research‐based solution to improve natural selection understanding among elementary school students while also validating the domain‐specific instruction theories underlying such learning processes. Consequently, two products result from our research: a transdisciplinary problem‐based learning (PBL) activity and new insights into elementary students’ understanding of natural selection and their learning processes in light of Malthus’ principle. In this process, we joined the efforts of primary school teachers, researchers in science and mathematical education, and an evolutionary biologist.

**FIGURE 1:**
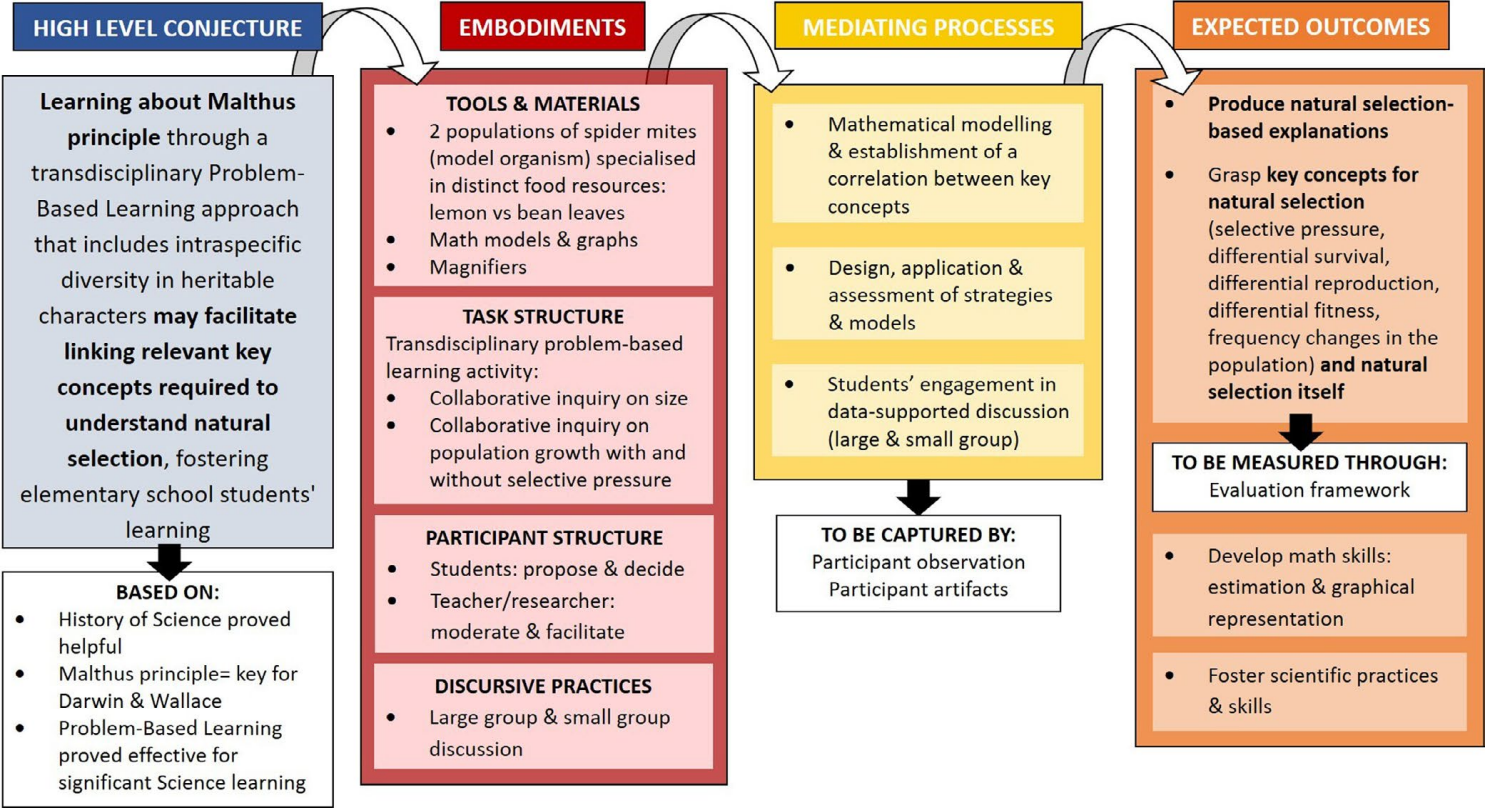
Conjecture map of our design research, adapted from Sandoval (2014). Based on prior research, we suggest that elementary school students’ understanding of natural selection could be fostered through a transdisciplinary problem‐based activity that includes exploring Malthus’ principle and intraspecific diversity in heritable characters (high‐level conjecture). Therefore, we designed a task consisting of a collaborative inquiry where students would explore population growth with and without selection pressure using mites as the model organism (embodiments). When engaging in this task, students design, implement, and evaluate different mathematical models of population growth while observing/analyzing the effects of the different factors involved and linking the historical key concepts (mediating processes). This should help them to better understand natural selection and allow them to produce natural selection‐based explanations. Also, they would improve their math and science skills (expected outcomes)

To ensure transparency and clearness of the design process, Sandoval (2014) recommends using a "conjecture map." Conjecture maps include 1) the embodiments (i.e., tools, materials, discursive practices) to be used in the intervention; 2) the mediating processes that such embodiments are meant to trigger; and 3) the expected outcomes to be achieved as a result of the emergence of such reasoning processes. Therefore, these maps capture the reasoning process of the researchers themselves, by making explicit how each element involved in the design relates to others, and, ultimately, serve to their research goals. Figure 1 introduces our conjecture map, which presents a summary of our design and how its various elements relate to each other. The results presented in this paper only correspond to the first cycle of the design and application of this intervention.

### 2.1 Design and implementation of the educational intervention

#### 2.1.1 Basic principles guiding our design

The ability to use natural selection to explain or predict biological situations requires students to understand, articulate, and put in action several key concepts. Notably, many researchers in evolution education have listed some of the distinct key concepts involved (Anderson et al., 2002; Nehm & Ridgway, 2011; Tibell & Harms, 2017). We will follow the list of key and threshold concepts recently proposed by Tibell and Harms (2017), who considered published lists of key concepts and then summarized and organized them into main principles. Furthermore, they proposed key concepts that are more generalizable and less sensitive to the surface features of a situation/problem. One such example is the key concept of “selective pressure,” which replaces other less generable key concepts such as “competition” and “limited resources,” which were presented in Anderson et al. (2002) and Nehm and Ridgway (2011) and merely represent some of the many selective pressures that can cause evolution by natural selection. Finally, unlike Anderson et al. (2002) and Nehm and Ridgway (2011); Tibell and Harms (2017) included differential reproduction as one of the key principles of evolution by natural selection. This is particularly important since differences in fitness among individuals are determined by the differences in their contributions to the next generations’ gene pool (Orr, 2009).

To identify which key concepts from the list of concepts by Tibell and Harms (2017) were acknowledged by Darwin as crucial in his development of the theory of natural selection, we searched for evidence in both Darwin's biography (Barlow, 1958) and his initial descriptions of evolution by natural selection (Darwin & Wallace, 1858). This comparison is presented in Tables 1 and Table S1 in Appendix S1.

**TABLE 1:** List of the key concepts of evolution by natural selection (from Tibell & Harms, 2017) and how have these been addressed in the activities and biological scenario presented to students in the evaluation framework

Principles	Key concepts (KCs)	How this KC is addressed in the activity	How this KC is addressed in the evaluation framework
Variation	KC1: Origin of variation (genetic changes)	Although we present a species with variable traits, the genetic basis of these is not discussed further than the traits being heritable	Although we present a species with variable traits, the genetic basis of these is not discussed further than the traits being heritable
	KC2: Individual (phenotypic) variation	Sessions 2 and 3: Spider mites populations differ in their ability to feed on distinct food sources	Butterflies differ in their ability to feed on distinct food sources
	KC3: Differential fitness (likelihood to survive and reproduce)	Session 3: Individuals of the two populations of spider mites differ in their probability of surviving and reproducing in the described environment	Individuals of the two varieties of butterflies differ in their probability of surviving and reproducing in the described environment
Reproduction	KC4: Heritable traits	Sessions 2 and 3: The ability of mites to feed from distinct food sources is a variable trait that passes from parents to offspring	The ability of butterflies to feed from distinct food sources is a variable trait that passes from parents to offspring
	KC5: Reproduction	Session 2: Each adult female lays 100 eggs and dies soon thereafter. From these eggs, 100 individuals are born (half males, half females)	Each adult butterfly lays four eggs and dies soon thereafter. From these eggs, four individuals are born
Selection	KC6: Selection pressure	Session 3: Resource availability imposes a selective pressure on the mite population, thereby limiting population growth. This selective pressure was distinct for the two distinct mite populations	Resource availability imposes a selective pressure on the butterfly population, thereby limiting population growth. This selective pressure was distinct for the two distinct butterfly varieties
	KC7: Differential survival	Session 3: In the described environment the mites that can feed from lemon tree leaves had increased probability of survive, when compared to those that feed on bean leaves.	In the described environment, butterflies that can feed from flowers with a long calyx had an increased probability of survival when compared to those that feed from flowers with a short calyx
	KC8: Differential reproduction	Session 3: In the described environment, the mites that can feed from lemon tree leaves had increased probability of reproduce, when compared to those that feed on bean leaves.	In the described environment, butterflies that can feed from flowers with a long calyx had an increased probability of reproducing when compared to those that feed from flowers with a short calyx
	KC9: Frequency change	Session 3: In the context presented in session 3, the mites that can feed from lemon tree leaves survive more have a higher probability of survival and have more offspring than those that feed from bean leaves. Over generations, this results in a higher frequency of the lemon tree population	In the environment presented in the figure, butterflies that can feed from flowers with a long calyx have a higher probability of survival and have more offspring than those that feed from flowers with a short calyx. Over generations, this results in a higher frequency of the variety with long proboscides
	KC10: Speciation	Not addressed	Not addressed

Notably, we found evidence supporting the notion that Darwin articulated and put in action, most of the key concepts (KCs) proposed by Tibell and Harms (2017), with the exception of two key concepts: speciation and the (genetic) origin of variation (Table 1). Regarding the genetic origin of variation, although Darwin mentions that “*during millions of generations, individuals of a species will be occasionally born with some slight variation*” (Darwin & Wallace, 1858 p.52), he was unaware of the genetic basis and mechanisms behind these variations. Accordingly, the origin of variation was not addressed during the planned educational activity. Although we present a species with variable traits in our activity, the genetic basis of these traits was not discussed further than the traits being heritable. Despite Darwin mentioning speciation in his initial 1857 letter to Asa Gray (Darwin & Wallace, 1858) in his autobiography, he identifies this discovery as occurring later than the discovery of the process of natural selection (Barlow, 1958). Accordingly, we do not address this concept in the educational activity.

Malthus’ principle is based on mathematical models that describe population growth as a function of resource availability. Therefore, we aimed to design a transdisciplinary activity that would require mathematical and biology skills and knowledge to be solved. By designing an interdisciplinary activity that simultaneously explores natural selection and mathematical learning goals, we aimed to i) link biology and maths disciplines and allow students’ engagement in mathematical thinking and the development and use of models, which are two scientific practices that students are expected to learn (National Research Council, 2012); ii) allow elementary school teachers to include evolution in teaching—even if this topic is not explicitly included in the learning goals of their national curriculum—to increase the likelihood of this concept being explored in these school grades. To further align our didactical proposal with the learning goals typically explored in elementary school classes, we aimed to design activities that further engage students in scientific practices included in Portuguese science standards (Portuguese Government/Ministry of Education, 2018a, 2018b, 2018c, 2018d) and those of other nations (National Research Council, 2012, National Research Council, 2007; Greek Government Gazette 303Β/13‐03‐2003).

To engage the students in the activity, we aimed to have at least one practical activity that would promote contact with animals since exploring real animals (either through direct contact or through films) was shown to increase students’ interest and competence (Hummel & Randler, 2012). To achieve this, we choose the two‐spotted spider mite (*Tetranychus urticae*)—an agricultural pest—because i) it was easily brought to the classroom for the students to observe and “manipulate”; ii) *T. urticae* displays intraspecific variability, with different populations being adapted to different host plant species (Migeon et al., 2011), what can result in fitness differences under selective pressures that are easily manipulated by the amount of food of each type provided; iii) has a short life cycle (generation time of approximately 13 days), which allowed us to follow evolution over short time scales because “deep time” has been proven to be a difficult concept for many groups (Catley & Novick, 2009; Cotner et al., 2010); and iv) individuals of this species are highly fecund, with females laying up to 10 eggs per day over a period of 20–30 days (Wrensch & Young, 1975); consequently, populations experience exponential growth and quickly deplete their resources, making them ideal for exploring Malthus’ principle.

##### The proposed didactic sequence

Our PBL activity (for more information on PBL and its potential in education see review at Hmelo‐Silver, 2004) consisted of three sessions of 150 min each. With the support of the students’ teacher in each class, the three sessions took place within one week and were led by research team members experienced in teaching these grades. The aims of each session are detailed in Table 2.

**TABLE 2:** Biology and mathematics learning goals for each of the three sessions

Session	Learning goals	
	Biology	Mathematics
1	Scientific instrument manipulation skills Designing solutions for problems Exploring spatial scales	Length measurements Scales Mathematical problem‐solving skills
2	Individual phenotypic variation (KC2) Heritable traits (KC4) Using mathematical thinking and modeling to estimate population growth due to reproduction (KC5)	Algebraic operations Geometric progressions Graphic representation of data Mathematical problem‐solving skills
3	Resource availability (KC6) Differential survival (KC7) and reproduction (KC8) Frequency changes due to evolution by natural selection (KC9) Using mathematical thinking and modeling to estimate population growth considering individuals’ fitness (KC5 and KC3)	Algebraic operations Geometric progressions Graphic representation of data Mathematical problem‐solving skills

Abbreviation: KC, key concepts.

The targeted concepts and sessions in which these were explored are described in Table 1.

###### Session 1

In the first session, we introduced the model species and students were asked to solve mathematical problems related to size measurements and scales. This allowed students to explore spatial scales, a threshold concept important for evolution understanding according to Tibell and Harms (2017) (Table 2). Students were asked to individually draw and share what they thought a mite looked like with the class, which uncovered previous conceptions about this species. Students were then invited to observe spider mites using various instruments, without being informed of the magnifications used, so they could collaboratively propose strategies to estimate the size of the spider mites using mathematical thinking. After solving this mathematical problem, students’ initial conceptions were compared to their observations.

###### Session 2

During the second session, students were introduced to the research group MITE2: Multidisciplinary Investigation Targeting Ecology and Evolution from the Centre for Ecology, Evolution and Environmental Changes based at the University of Lisbon (https://ce3c.ciencias.ulisboa.pt/sub‐team/mite2) through a short movie. This research team provided the spider mites used in the activities and the movie guided the students through their laboratories and introduced some of their research projects using this organism. Besides introducing students to an example of how researchers work, the video allowed us to provide a real context for the problem posed to students.

Students were informed that two individuals of one spider mite population that feeds on citrus tree leaves (henceforth referred to as the lemon specialist) and six individuals of another spider mite population that feeds on bean plant leaves (henceforth referred to as the bean specialist) would be sent by the MITE2 research group on that day to be presented to other classes for observation and to perform more experiments. The teacher of the class divided students into smaller groups (between 4 and 6 members), and these groups were asked to work collaboratively to propose strategies to mathematically model the growth of the population and to estimate and graphically represent the number of lemon specialists that were expected to exist in 45 days. The entire class discussed what information regarding species’ biology would be needed. After reaching a consensus on the information needed to solve the mathematical problem—and to simplify the mathematical modeling—students were told to consider a sex ratio of 1:1, a generation time equal to a life expectancy of 15 days, and that each female lays approximately 100 eggs, from which 100 individuals are born. Using the aforementioned parameters, students discussed the best strategy to solve this problem in smaller groups and applied it to estimate the solution. Each group then presented the strategy they used and the results they obtained to the class, and all students ultimately discussed and decided on the best strategy to be applied. Each group was asked to estimate, using this method, the number of bean specialists within 45 days and to graphically represent the number of mites of each plant specialist for each 15‐day period. While solving this problem—applicable to both plant specialists—students were expected to explore the mites’ reproduction (KC5, Table 1) by estimating and graphically representing the geometric population growth expected under an unlimited resource scenario (no selective pressure present). They explored this pattern for two mite populations (KC2, Table 1) that differ in their heritable ability to feed on distinct food sources (KC4, Table 1).

###### Session 3

During the third session, students were asked to do the same exercise as in the second session while considering the selective pressure (KC6, Table 1) imposed by resource availability: We could only provide 100 lemon tree leaves and 10 bean leaves per week to all the mites, which would be kept in a single large box. The number of leaves was chosen so the bean specialist, initially most frequent, would have fewer resources available to feed on, thereby changing its representation in the population (K9, Table 1). Once again, students decided what information they required regarding species biology and how could they use it to answer this question via an initial class discussion. Students were told that each leaf (regardless of the plant type) could feed a maximum of 100 mites in a week.

Again, the class teacher divided the students into small groups of 4 to 5 students. Students were then asked to propose a strategy to estimate the number of bean and lemon specialists on this limited resource scenario, 45 days later, building from the procedure developed in the previous session. At the end of the activity, students were asked to observe the results of their mathematical model, discuss it in their groups, and explain why the least frequent plant specialist had become the most frequent one (KC9, Table 1). Furthermore, they were asked to compare the results obtained in this scenario with those of the unlimited resource scenario and discuss the reasons for the observed differences. Solving the proposed tasks required that students understand that, in the proposed situation, resource availability (KC6, Table 1) resulted in distinct fitness (KC3, Table 1) between the two mite populations due to their differential survival (KC7, Table 1) and reproduction (KC8, Table 1).

### 2.2 Sampled classes

The 4th grade, in Portugal, is the end of the first cycle of basic education (1st CEB, from the 1st to the 4th grade). The 1st CEB in Portugal has particular features: i) It is the first cycle of mandatory education as kindergarten is not mandatory; ii) during the first years of this cycle students learn how to read and write; ii) there is a single teacher who teaches all the subjects (Portuguese, Mathematics, Study of the Environment, Artistic education and Physical Education; Gabinetes da Secretária and de Estado Adjunta e da Educação e do Secretário de Estado da Educação, 2018). Given our goal, we decided that the last grade of the 1st CEB was the most interesting to answer our research question, due to the complexity of the mathematical calculations needed for this activity which are only covered by the end of the first cycle. Two classes of fourth‐grade students (ages 9–10 years old) from two distinct schools engaged in the previously described didactic activity. The two schools were from the northern region of Portugal. This involved convenience sampling since schools were not chosen randomly (Cohen et al., 2007). Instead, they were chosen among those with which the research team had worked before in other classes and topics and that had at least two fourth‐grade classes. SA was a private school located in the center of a big city in the northern region of Portugal, while SB was a public school located in a more rural area, 20 km away from this city. According to publicly available information, most parents with children at SB only completed the 6th grade or below and 82% of the students were included in the 1º and 2º class of family support for social security due to their low family income. No information on parents’ academic or income levels was available for SA.

The class in SA had 19 students (henceforth referred to as the SAT class) and in SB 25 (henceforth referred to as the SBT class). No personal information about students was collected since their answers were identified by a code made from their student number, class, and school. Informed consent was obtained from the students’ parents, the school boards, and teachers before the implementation of the activity and test. The procedures followed were approved by the school boards and are in accordance with the ethical standards of the Ethics and Deontology Council of the University of Aveiro and with the Helsinki Declaration of 1975, as revised in 2008.

### 2.3 Design and application of the evaluation framework

To evaluate students’ understanding of evolution by natural selection, we adapted and applied an evaluation framework. In the following sections, we describe i) the evaluation instruments upon which we designed our framework; ii) the features of our evaluation instrument; iii) how the test was applied in the classrooms, and iv) the procedure used to evaluate and score students’ answers.

#### 2.3.1 Evaluation instruments upon which we designed our framework

When we started this project, two evaluation frameworks were available to evaluate elementary school students’ understanding of evolution by natural selection: the interview script used by Kelemen et al. (2014), and Emmons et al. (2017) and the test proposed by Sá‐Pinto, Pinto, et al. (2017). Although we could not find information on the preferences of elementary school teachers for performing student evaluations, our lengthy experience and contact with this school grade suggests that these mostly use written tests. To elaborate on an instrument that could also be useful and applied by teachers, we followed Sá‐Pinto, Pinto, et al. (2017); Sá‐Pinto, Cardia and Campos (2017) and designed a written test. We also retained some features of this framework that distinguishes it from the one used by Kelemen et al. (2014) and Emmons et al. (2017), namely i) the final outcome of the biological scenario was not provided to the students, thus allowing students to reveal fixist ideas; ii) unlike Kelemen et al. (2014) and Emmons et al. (2017); Sá‐Pinto, Pinto, et al. (2017); Sá‐Pinto, Cardia, et al. (2017) did not ask students any isolated fact questions regarding the trait inheritance, trait constancy, survival, or reproduction ability of each phenotype before or after asking them to predict the outcome of the biological scenario to avoid influencing students’ predictions and justifications; iv) like in Sá‐Pinto, Pinto, et al. (2017); Sá‐Pinto, Cardia, et al. (2017), students were informed that the two phenotypes in the test were heritable—without this information, it would be impossible to evaluate how much the phenotypic differences could result from environmentally driven morphological plasticity.

#### 2.3.2 The test and its implementation with students

The instrument used as pre and post‐test presented students with a biological scenario similar to the one explored in the educational activity (Table 1): i) an isolated population of butterflies (mites in the activity); ii) with a variable and heritable character with two distinct phenotypes that influenced their ability to feed on two distinct food resources (i.e., butterflies with long or short proboscises feeding on flowers with long and short calyxes; bean and lemon specialists eating bean or lemon leaves in the activity); iii) the most frequent phenotype would have fewer resources available to feed on (in the activity the bean specialists in a box with more lemon leaves than bean leaves). The test is presented in detail in Figures S1 and S2 of the Appendix S1.

Students were asked to think forward in time and predict the outcome of this scenario and then describe how the butterfly population would look in 100 years. The test was read aloud to the class, and students were asked to write a justified prediction and draw it. After finishing these tasks, each student was individually asked to verbally explain her/his predictions and justifications to the researcher and, when the student provided more information at this stage, she/he was asked to complete her/his written answer in the test form. No corrective feedback or additional information was provided by the researcher during this phase. For students with writing difficulties, the answers were provided verbally and registered by the researcher using the students’ exact words. This procedure was followed independently of the type of predictions and justifications put forward by the students. In total, between 20 and 30 min were required to obtain all of the students’ answers for each class. This evaluation procedure was applied immediately before (pretest) and approximately 20 days after the activity was performed (post‐test).

#### 2.3.3 Procedure to evaluate students’ answers and score the evaluation criteria

To evaluate students’ answers, we used criteria developed by other authors (Kelemen et al., 2014; Sá‐Pinto, Cardia, et al., 2017; Sá‐Pinto, Pinto, et al., 2017) in the context of the aforementioned framework. These were complemented with the inclusion of another criterion that targets whether students’ predictions integrate information about the selective pressure: resource availability. These criteria formed the items of our rubric. The complete definitions of each rubric item are provided in Table S2 in Appendix S1. These rubric items allowed us to classify answers according to the student's type of prediction (i.e., fixist, fittest, or equilibrium) and the justification provided (i.e., developmental, teleological, resource availability, differential survival, or differential reproduction).

- *Fixist* answers predicted that the initially most common (and less fit, if no other biological meaningful justification was provided) phenotype would remain the most common in 100 years;
- *Fittest* answers predicted that the fittest phenotype would become the most frequent in 100 years (predicting a strong frequency change KC9, Table 1);
- *Equilibrium* predictions stated that both phenotypes would become equally frequent in 100 years (predicting a moderate frequency change KC9, Table 1).

The level of understanding of evolution by natural selection (LUENS) revealed by each answer was determined by the sum of the scores attributed for each rubric item identified in that answer, regarding both predictions and corresponding justifications. We attributed a score of 1 to the rubric items *resource availability* (selective pressure KC6, Table 1) and *differential survival* (KC7, Table 1). A score of 2 was attributed to *differential reproduction* (selective pressure KC8, Table 1) since this better correlates with individuals’ contributions to the gene pool of the next generation (i.e., individuals’ fitness). To determine the score of each type of prediction, we estimated Spearman's correlation coefficient (and its corresponding statistical significance) between them and the rubric items related to evolution (namely *resource availability, differential survival* and *differential reproduction*). These results, depicted in Table 3, mostly confirm those obtained in previous studies (Sá‐Pinto, Cardia, et al., 2017; Sá‐Pinto, Pinto, et al., 2017), showing positive and significant correlations between *fittest* predictions and justifications mentioning *resource availability, differential survival* and *differential reproduction* and negative and significant correlations between these three rubric items and *fixist* predictions. While the results of previous studies (Sá‐Pinto Cardia & Campos, 2017; Sá‐Pinto, Pinto, et al., 2017) showed that *equilibrium* predictions were negatively and significantly correlated with justifications mentioning *resource availability, differential survival,* and *differential reproduction*, no significant correlation was found in the present study. This suggests that students providing *equilibrium* predictions are not relating the frequency changes with biological important parameters, nor thinking evolutionarily. Based on these results, we attributed a score of 1 to *fittest* predictions and a score of 0 to *fixist* and *equilibrium* predictions. All other rubric items received a score of 0. Given this score rating, LUENS can range between 0 (for answers with no evidence of evolution understanding) and 5 (for answers with evolutionary predictions justified by all components of the key concepts important to understanding natural selection). The present framework evaluates whether students can apply all KCs related to the principle of selection (Tibell & Harms, 2017; Table 1)—except for speciation since this KC was not addressed in this activity for the aforementioned reasons.

**TABLE 3:** Spearman's correlation coefficient and the statistical significance obtained between distinct types of predictions and rubric items related to evolution in students’ justifications

Prediction type	Resource availability	Differential Survival	Differential reproduction
*Fixist*	−0.672**	−0.398**	−0.329**
*Equilibrium*	0.010	0.082	−0.046
*Fittest*	0.873**	0.515**	0.465**

** statistically significant at *p* < .01

For a detailed explanation of how students’ answers were coded, see examples in Figure S3 in Appendix S1 and Table S2 in Appendix S1.

#### 2.3.4 Ensuring the validity of the evaluation instrument

To ensure that the chosen evaluation instrument was valid, we i) designed our instrument by adapting a previously published instruments (Sá‐Pinto, Cardia, et al., 2017; Sá‐Pinto, Pinto, et al., 2017), ii) ensured that all key concepts required for evolution understanding (Tibell & Harms, 2017) that were explored in our activity were present in our evaluation instrument (Table 1), and iii) studied the correlation between the students’ predictions and justifications to decide on the scoring procedure. Furthermore, we applied the same test procedure in two control classes, which were classes from the same schools in which we did not apply the aforementioned activity or any evolution‐related activity (henceforth referred to as SAC [*N* = 21] and SBC [*N* = 19]) on the same days that the target classes were tested. Target and control classes were chosen by the school director and teachers based on their availability according to the school schedule. We used control classes to check for the impact of the double exposure of students to our test and to evaluate the internal validity of the process (Lahm, 2004). The pre‐ and post‐tests of the two control classes (SAC and SBC) did not significantly differ (*Z*_SAC_ = −0.447, *p* = .655 and *Z*_SBC_ = −1.604, *p* = .109), thus confirming the internal validity of the process (Lahm, 2004). Finally, two in dependent researchers—one evolutionary biologist with a background in science education and one elementary school teacher, evaluated all the students’ answers. Interrater reliability was estimated as the percentage of the initial agreement between raters (McHugh, 2012). Answers not equally rated by the two researchers were discussed, and, if a consensus could not be reached, these were removed from the analysis. Since interrater reliability was >89% for all analyzed items, the reliability of this procedure was considered acceptable (Stemler, 2004, p.2).

### 2.4 Data analysis

McNemar and Wilcoxon tests were used to estimate the statistical significance of, respectively, changes in the frequency of each rubric item and students’ LUENS between pre‐ and post‐tests. All statistical analyses were performed using SPSS v23. The database housing the results of the students’ answers analysis is deposited in the Dryad repository https://doi.org/10.5061/dryad.n2z34tmww.

To complement the data collected from students’ test answers and characterize the learning processes that occurred in the target classes, we collected field notes during participant observation in the sessions, took photographs of students’ productions and recorded their discussions. These documents were used to describe the students’ learning process and document how they explored and linked the target key concepts during the sessions.

## 3 RESULTS

### 3.1 Evidence of the mediating processes during the educational activity

#### 3.1.1 Students’ engagement and conceptual field building

During the three sessions, students were actively engaged in the proposed tasks (see examples of students’ engagement in the tasks in Figures S4 to S6 in Appendix S1). They used the materials provided to them and collaboratively (in both small and large groups) proposed, discussed, implemented, and revised solutions for the problems and identified the parameters important for population growth, mathematical modeling, and calculation strategies to estimate population sizes. In both large and small groups, they also graphically depicted the results. Moreover, they further discussed these results in the large group. A description of students’ solutions, discussions, and productions in each session can be found in the section *Mathematical modelling and linkage to historically important key concepts in* Appendix S1.

As planned, in session 2 students explored and linked the key concepts KC2, KC5, and KC4 (as defined in Table 1) and KC2, KC3, KC4, KC5, KC6, KC7, KC8, and KC9 (as defined in Table 1) in session 3 (see description of the sessions in the section *Mathematical modelling and linkage to historically important key concepts* and Figures S4 to S6 in Appendix S1).

### 3.2 Evaluation of the impacts of the activity in students’ LUENS

**FIGURE 2:**
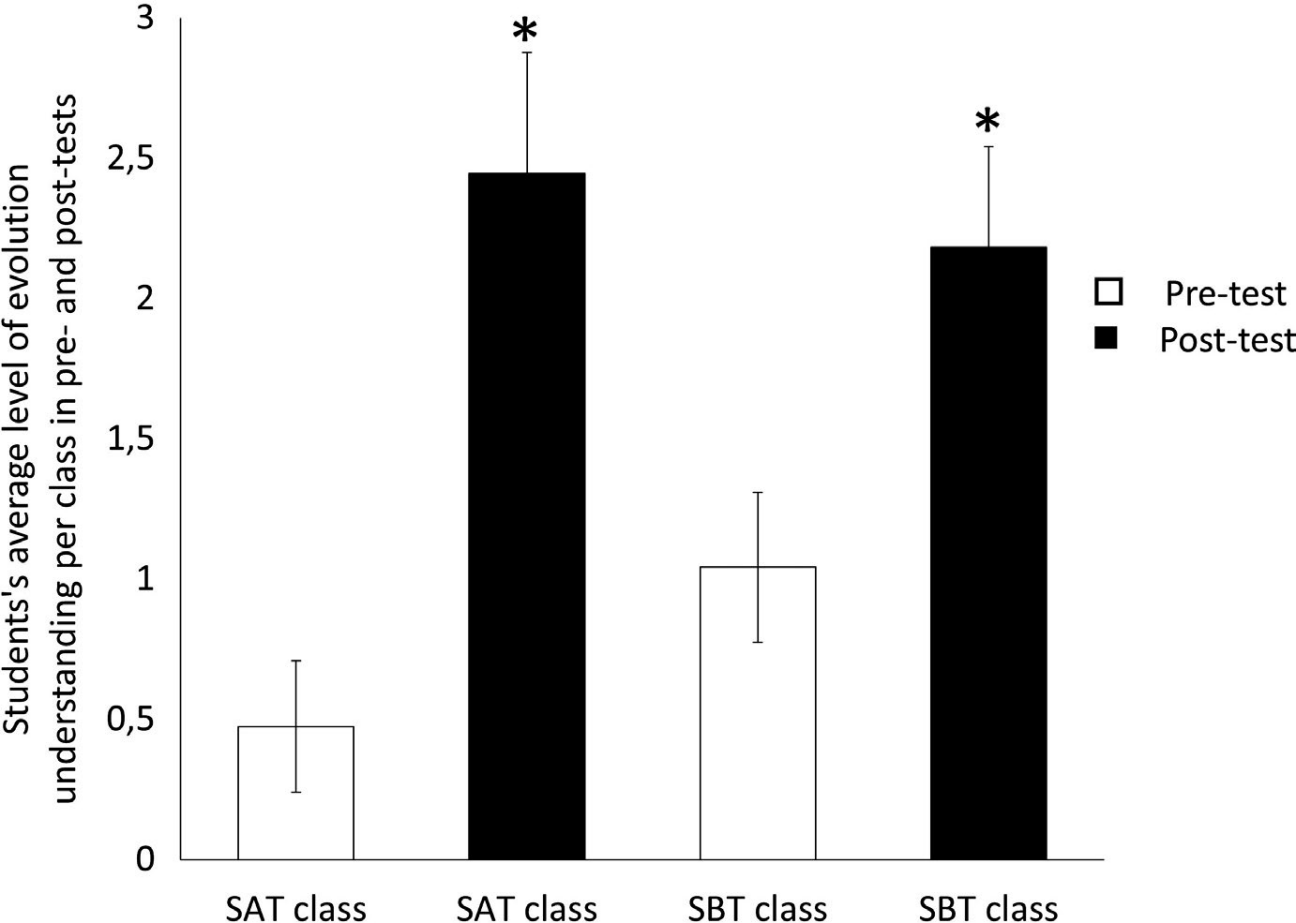
Average level of understanding of evolution by natural selection (LUENS; maximum level 5, based on the evolution understanding evaluation framework) revealed by students’ answers in pre‐ and post‐tests in target classes. White and gray bars indicate pretests and post‐tests, respectively. * indicates a value significantly different from the one obtained by the students in pretests according to Wilcoxon test results (*p* < .05). Vertical lines represent standard errors of the mean. SAT, School A target class; SBT, School B target class

The impact of our proposed activity was examined in the two target groups that we applied the activity with (SAT and SBT). Significant differences in LUENS (Z_SAT_ = −2.961, *p* = .003 and Z_SBT_ = −2.591, *p* = .010) were recorded between the pre‐ and post‐tests in the two target classes, with post‐tests revealing a better understanding of evolution (Figure 2).

**FIGURE 3:**
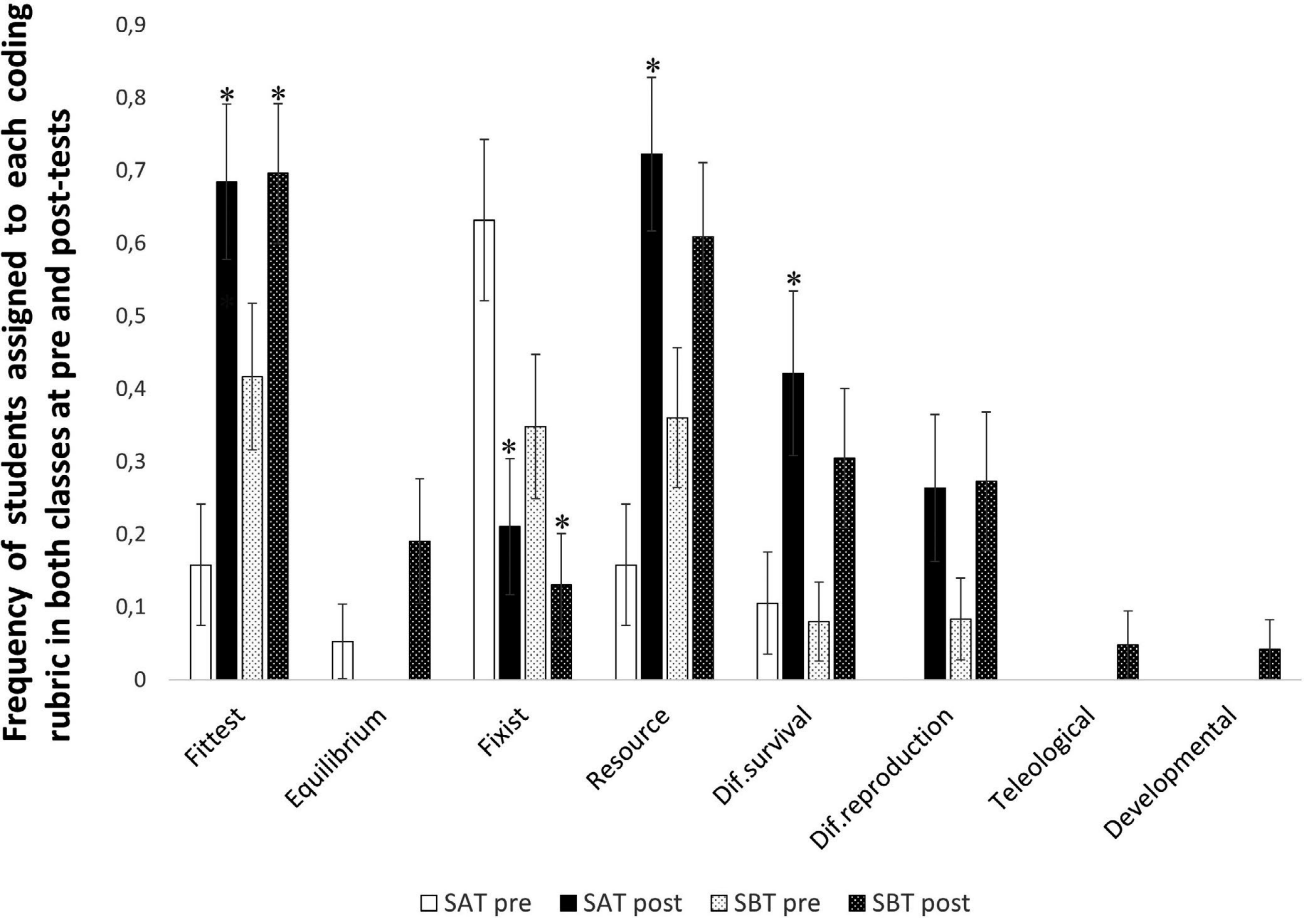
Frequencies of students’ answers assigned to each coding rubric item in pre‐ and post‐tests. White bars indicate the SAT class pretest. Black bars indicate the SAT class post‐test. White dotted bars indicate the SBT class pretest. Black dotted bars indicate the SBT class post‐test. Asterisks (*) denote post‐test values that significantly differ from pretests according to McNemar test results (*p* < .05). Vertical lines represent the standard errors of the difference between two proportions

The percentage of students’ answers falling under the category of each rubric item is presented in Figure 3 (and Table S3 in Appendix S1). Differences between pre‐ and post‐tests were observed in *i*) the type of prediction made by the students and *ii*) the justification of this prediction. A significant increase in *fittest* predictions and a significant decrease in *fixist* predictions were observed between pre‐ and post‐tests in both target classes (*p* < .05).

At the pretest, 63.4% of the students in the SAT class provided *fixist* predictions, with *fittest* predictions being the second most frequent (15.8%, Figure 3 and Table S3 in Appendix S1). However, in the SBT target class, students mostly provided *fittest* predictions (41.7%), with *fixist* predictions being the second most frequent (34.8%, Figure 3 and Table S3 in Appendix S1). *Equilibrium* predictions were the least frequent in all classes (5.3% in SAT and 0% in SBT; Figure 3 and Table S3 in Appendix S1). In post‐tests, the *fittest* predictions increased and became the most frequent in both classes (68.4% in SAT and 69.6% in SBT; Figure 3 and Table S3 in Appendix S1). Notably, many of the *fixist* predictions were justified with a mathematical model for population growth that only accounts for the number of offspring an individual can have (see, e.g., Figure S3a in Appendix S1). The changes observed to the *fittest* predictions involved students introducing additional biological parameters to this model, namely resource availability and the consequent differential survival and reproduction of individuals in the diverse population. But despite the observed increase in the frequency of students justifying their predictions with *resource availability*, *differential survival* or *differential reproduction* of the phenotypes in post‐tests, (Figure S3 in Appendix S1 and Table S2 in Appendix S1 e.g., Table S3 in Appendix S1 for frequencies and Figure 3 for a graphical representation) these differences were statistically significant only in the target SA school, and only for the items *resource availability* and *differential survival* (*p* = .002 and *p* = .031, respectively; Figure 3 and Table S3 in Appendix S1).

*Teleological* and *developmental* justifications were rare in both classes for both pre‐ and post‐tests, and no significant differences between pre‐ and post‐tests were observed for these two types of justifications in any of the classes (see frequencies in Table S3 in Appendix S1 and examples in Table S2 in Appendix S1).

## 4 DISCUSSION

The results of the present study indicate that our approach allowed elementary school students to explore and link all of the historically important key concepts. Notably, this approach was able to promote elementary school students’ understanding of evolution by natural selection.

During session 2, students applied three of the eight historically important key concepts. In session 3, all eight of these concepts were applied to solve and discuss the results of the proposed problem (see description of the session in the section *Mathematical modelling and linkage to historically important key concepts in* Appendix S1). Moreover, using this approach led to a high and significant increase in students’ LUENS (average increase of LUENS of 1.51 on a scale from 0 to 5), which was mostly due to i) the significant increase of *fittest* predictions and the significant decrease of *fixist* predictions; ii) the strong and statistically significant increase of justifications mentioning the *resource availability* and *differential survival* in the case of SAT (Figure 3).

Our results support the hypothesis that PBL activities designed to explore concepts and conceptual fields that were important during the historical process of scientific discoveries foster science understanding in students. The history of science has been widely used to design activities that allow students to learn about the nature of science and develop important scientific and critical thinking skills (Clough, 2010; Gooday et al., 2008; Mavrikaki & Kapsala, 2014). Regarding evolution, many textbooks mention the important contribution of Malthus’ principle for developing the concept of natural selection (see, e.g., Silva et al., 2004; Mader, 2009). However, to the best of our knowledge, no educational activities have been designed for students to link the concepts underlying this principle with those of intraspecific variability and resource use through active learning. Our results are promising and highlight the potential of applying educational activities designed to promote historically important conceptual fields about evolution.

It is interesting to note that in pretests, many students that provided fixist explanations based these predictions on simple mathematical models that only consider a few parameters (namely the initial proportions of the varieties (KC2), trait heritability (KC4), and, in some cases, the potential reproductive output of the species (KC5); see Table S2 in Appendix S1 and Figure S3 in Appendix S1 for examples). In fact, the observed improvement in LUENS was achieved because students accounted for other biologically meaningful parameters (and evolution key concepts) in their answers, especially the selective pressure imposed by the available resources (KC6, Table 1) and the resulting differential survival (KC7, Table 1) and reproduction of the distinct populations (KC8; Table 1), which allowed them to predict the frequency change (KC9; Table 1). During the activity, these concepts were linked through increasingly complex mathematical models that incorporated several meaningful biological parameters and were collaboratively built by the students to solve the real‐life problem posed to them. This further supports the potential of educational transdisciplinary activities that use mathematical modeling to promote and support science learning (see review in National Research Council, 2007).

Other features of our activity also likely contribute to its success, namely i) the engagement of students with real organisms that they have observed and measured (Broder et al., 2018); ii) the context of the activity was a real‐life problem (i.e., the need to grow mites in order to repeat the activity in other schools); iii) the cooperative PBL approach followed, with repeated cycles of learning and knowledge application; iv) the short life cycle of the mites, which would allow evolution to be observable in a very short period of time. We acknowledge that the model organism we used and the contact with the research team may not be easy to replicate in some schools. This could be a limitation for teachers who wish to apply this activity in their schools. However, this limitation might be easily overcome by using other organisms that have already been explored in schools. For instance, despite its longer life cycle (one year), the silk moth (*Bombyx mori*) has great reproductive potential and is heavily dependent on a specific type of food, which rapidly becomes a limiting resource. In this scenario, students can be asked what would happen if one individual is born with a heritable difference in its ability to eat other types of food.

Although other activities have been reported to explore natural selection with elementary school students (see, among others, Berti et al., 2017; Frejd et al., 2020; Kelemen et al., 2014; Sá‐Pinto, Cardia, et al., 2017; Sá‐Pinto, Pinto, et al., 2017; Shtulman et al., 2016), to the best of our knowledge, no other activity has engaged students in mathematical modeling to achieve this type of goal. However, mathematical thinking and the ability to develop and use models have been recognized as important scientific practices that students should learn since their initial years of schooling (National Research Council, 2012). When considering evolution, the ability to think mathematically while using and extending Malthus’ mathematical model on population growth by including other biological parameters was fundamental for Darwin and Wallace to reason about natural selection and, according to our results, may also influence students’ learning about this evolutionary process.

Among all of the important evolution‐related KCs required to understand the principles of selection (Tibell & Harms, 2017; Table 1), *differential reproduction* (and consequently *fitness* from the principle variation) was least commonly applied by students to justify their predictions in both tests. Moreover, although there was an increase in the frequency of its use from pre‐ to post‐test, this difference was not statistically significant. Our results are in line with those obtained by Brown et al. (2020), who reported that 32% of students used this key concept after a storytelling intervention. These results suggest that additional effort should be made to increase students' understanding of and ability to apply this key concept. To achieve this goal, we propose extending session 3 by asking students to estimate (and graphically represent) the number of viable offspring per individual that were able to survive and reproduce for each generation. Additionally, an activity that explicitly asks students to link the different key concepts (e.g., a conceptual map) could contribute to scaffolding their conceptual field of evolution by natural selection. This exercise is expected to improve students’ perceptions of these two key concepts. Additional possibilities that allow students to explore the importance of *differential reproduction* to drive frequency change involve the use of activities that directly explore sexual selection as the process driving reproductive success (Sá‐Pinto, Pinto, et al., 2017 for a review on the importance of sexual selection for evolution and evolution understanding as well as activities that aim to explore this process).

An interesting result from the present work is the low level of teleological justifications identified (<2% of the total number of answers). These results strongly contrast with those of previous studies with older students, which suggests that teleological thinking is one of the main difficulties precluding evolution understanding (see review in Galli & Meinardi, 2011). Many studies report a high frequency of misconceptions related to teleological thinking in older students, which are persistent and difficult to change—even through educational programs specifically designed to address them (Bishop & Anderson, 1986; Nehm & Reilly, 2007). Younger students were also shown to provide teleological explanations for biological scenarios involving natural selection before instruction (Brown et al., 2020). The causes for the differences between this and other studies are not yet clear and deserve further attention and studies comparing elementary students’ performance with distinct evaluation frameworks.

But a possible explanation for the low level of teleological explanations found in this and previous studies on evolutionary thinking (Emmons et al., 2017; Sá‐Pinto, Cardia, et al., 2017; Sá‐Pinto, Pinto, et al., 2017), when compared to those found in adults and older students (Bishop & Anderson, 1986; Miller et al., 2006; Nehm & Reilly, 2007; Prinou et al., 2011; Rutledge & Warden, 2000; Spiegel et al., 2012), could be the reinforcement of this misconception during people's lives. Several studies have suggested that teleological thinking in evolution can be reinforced by teachers, books, the media, and even by the way evolutionary biologists speak about evolution (Nehm et al., 2010; Prinou et al., 2011). This would support the importance of an early introduction of students to evolutionary processes, which has been advocated by several authors (e.g., Nadelson et al., 2009; Wagler, 2010, 2012; Campos & Sá‐Pinto, 2013; Kelemen et al., 2014; Berti et al., 2017; Pires et al., 2016; Emmons et al., 2017; Sá‐Pinto, Pinto, et al., 2017; Sá‐Pinto Cardia & Campos, 2017; Brown et al., 2020; Frejd et al., 2020). As suggested by Emmons et al. (2017), early instruction on evolution may preclude the development and strengthening of misconceptions on the topic, thereby providing children with scientifically accurate explanations to compete with inaccurate ideas in multiple learning and reasoning contexts. Further support to this idea comes from the work of Brown et al. (2020). The results from these authors suggest that teleological reasoning in elementary school students may be easy to overcome with instruction, a pattern that contrasts with what has been reported for older learners and adults (Bishop & Anderson, 1986; Nehm & Reilly, 2007).

In Portuguese official curricula, evolution by natural selection is not present as a learning goal until the 11th grade. Therefore, it is highly improbable that the students who engaged in our activity had previously explored this process in school. Both the present work and the work previously published on these grades (Berti et al., 2017; Brown et al., 2020; Emmons et al., 2017; Frejd et al., 2020; Kelemen et al., 2014; Sá‐Pinto, Cardia, et al., 2017; Sá‐Pinto, Pinto, et al., 2017; Shtulman et al., 2016) only evaluated the impact of students’ engagement in one activity exploring natural selection. However, as suggested by both Nehm (2018) and Vergnaud (2009), a clear understanding of natural selection and its key concepts (or “invariables”; Vergnaud, 2009) can only be achieved through the exploration of this process in distinct situations. Therefore, future studies should attempt to understand how addressing natural selection under distinct situations contributes to elementary school students’ understanding of evolution by natural selection.

## 5 CONCLUSIONS

In the present work, we present an innovative and effective approach to explore natural selection and promote evolution understanding in elementary school students. To foster learning about evolutionary processes, we designed a transdisciplinary activity that uses real‐world problems to engage students in mathematical modeling that links concepts that were historically important to Darwin discovering the process of natural selection. Our activity allowed students to put in action all the historically important key concepts and resulted in a significant increase in their understanding of evolution by natural selection. Despite this, the activity did not significantly increase students’ ability to use the key concept of differential reproduction, which suggests that this is a proximal development zone that additional activities could improve. The in‐depth study of the activity implementation revealed that some fine‐tuning of the activity may further enhance learning about this key concept. In contrast to what has been reported for older students and adults, we observed an unexpectedly low level of teleological answers from elementary school students. Together, these results contradict the general assumption that young children are unable to learn evolution by natural selection and mostly apply teleological thinking to biological processes. This result highlights the importance of early learning about evolution and raises new research questions related to the development and use of teleological explanations during a person's life.

## CONFLICT OF INTEREST

The authors declare no competing interests.

## AUTHOR CONTRIBUTION

**Xana Sá‐Pinto:** Conceptualization (equal); Data curation (equal); Formal analysis (equal); Funding acquisition (lead); Investigation (equal); Methodology (equal); Project administration (lead); Resources (supporting); Supervision (equal); Validation (equal); Writing‐original draft (lead); Writing‐review & editing (equal). **Alexandre Pinto:** Conceptualization (supporting); Resources (supporting); Writing‐original draft (supporting); Writing‐review & editing (equal). **Joana Ribeiro:** Conceptualization (equal); Data curation (supporting); Investigation (equal); Methodology (equal); Resources (equal); Writing‐original draft (supporting); Writing‐review & editing (equal). **Inês Sarmento:** Conceptualization (equal); Resources (equal); Writing‐original draft (supporting); Writing‐review & editing (equal). **Patrícia Pessoa:** Data curation (equal); Formal analysis (equal); Investigation (equal); Writing‐original draft (supporting); Writing‐review & editing (equal). **Leonor** **Rapoula Rodrigues:** Formal analysis (equal); Resources (equal); Writing‐original draft (supporting); Writing‐review & editing (equal). **Lucía Vázquez‐Ben:** Methodology (equal); Validation (equal); Writing‐original draft (supporting); Writing‐review & editing (equal). **Evangelia Mavrikaki:** Formal analysis (equal); Funding acquisition (lead); Methodology (equal); Project administration (lead); Validation (equal); Writing‐original draft (supporting); Writing‐review & editing (equal). **Joaquim Bernardino Lopes:** Conceptualization (supporting); Methodology (equal); Validation (equal); Writing‐original draft (supporting); Writing‐review & editing (equal).

## Supporting information

Appendix S1

## Data Availability

The database housing the results of the students’ answers analysis is deposited in the Dryad repository ‐ https://doi.org/10.5061/dryad.n2z34tmww.
