# Supplementary material for: Following Darwin’s footsteps: Evaluating the impact of an activity designed for elementary school students to link historically important evolution key concepts on their understanding of natural selection
Source: Ecol Evol. 2021 Aug 25;11(18):12236–50. doi: 10.1002/ece3.7849 (PMC8462140; doi:10.1002/ece3.7849)
Supplement: Supplementary file 1 — Appendix S1 [file ECE3-11-12236-s001.docx]

**APPENDIX**

**Figure A1- Evaluation instrument presented to the elementary school students as pre and post-test.**

**
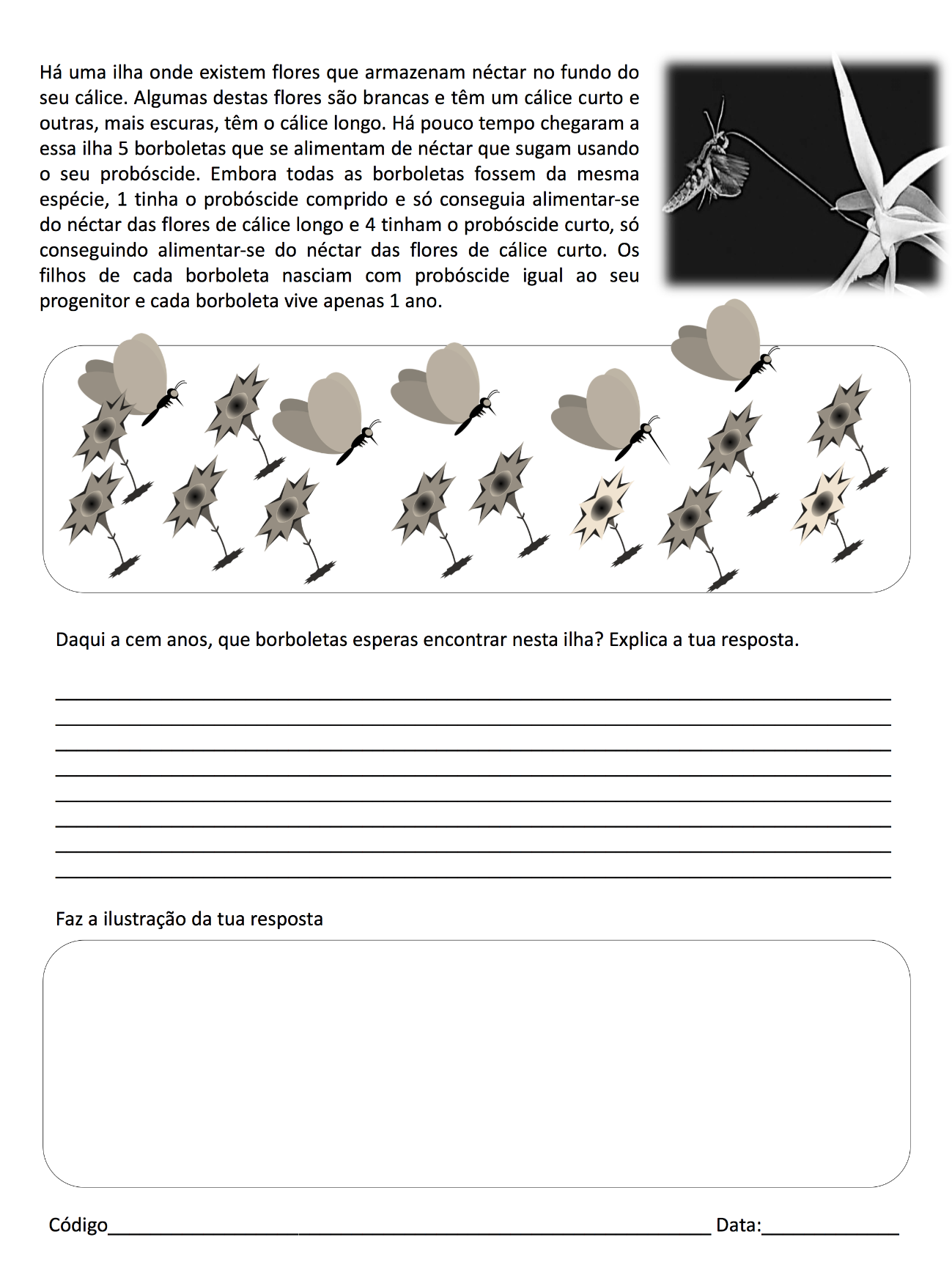
**

**Figure A2- English translation of the evaluation instrument presented to the elementary school students as pre and post-test.**

**
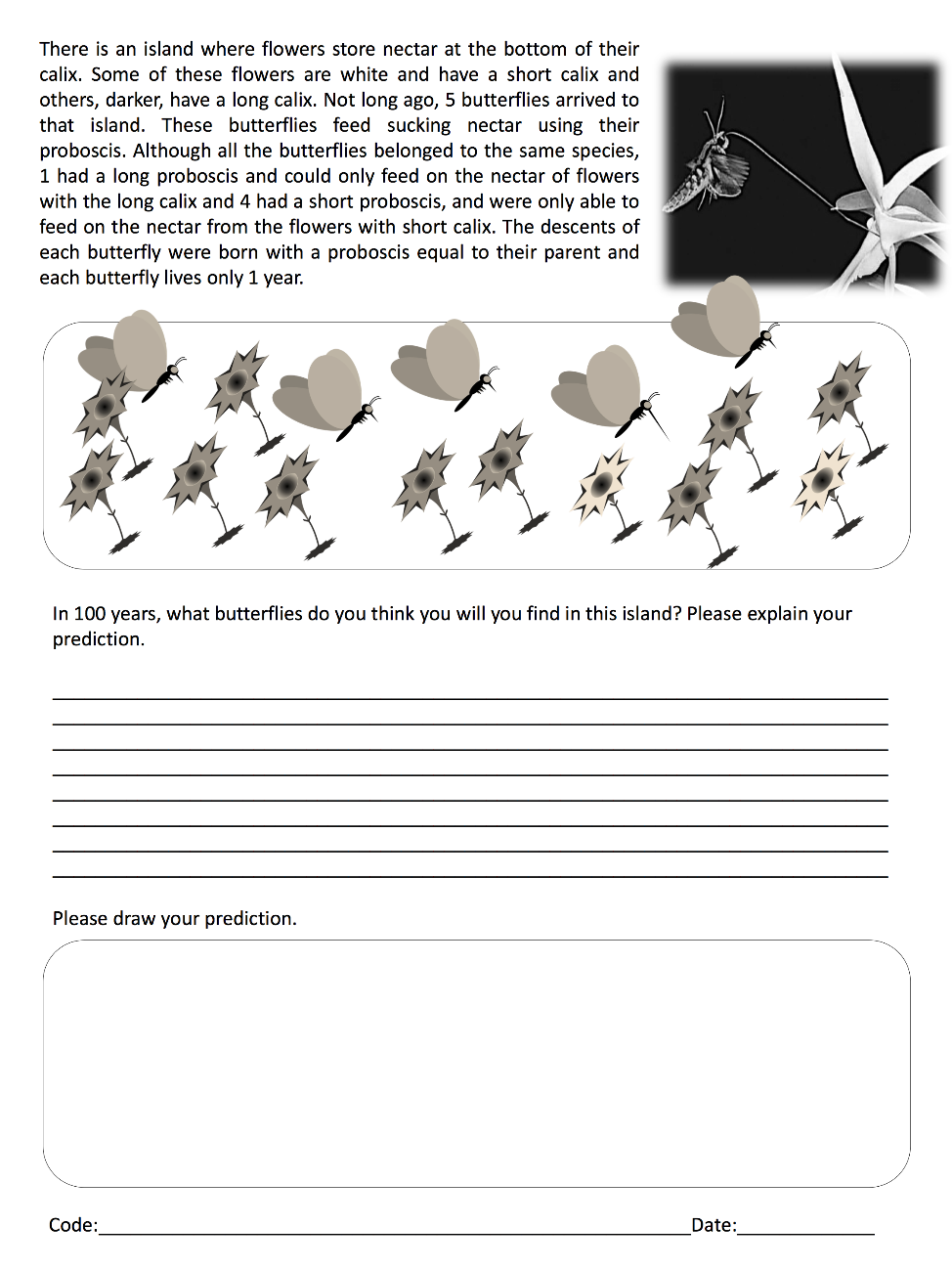
**

**Table A1- Evidence for the presence of** key concepts (KC) of evolution by natural selection (from Tibell & Harms, 2017) in Darwin’s initial publication on the process of natural selection (Darwin and Wallace, 1858) and in his diary (Barlow 1958)

| **Key concepts (KCs)** | **Evidence for this KC and how was it addressed by Darwin** |
| --- | --- |
| **KC1:**  Origin of variation (genetic changes) | *“In nature, we have some slight variation occasionally in all parts; and I think it can be shown that* ***changed conditions of existence is the main cause of the child not exactly resembling its parents****”* (Darwin and Wallace, 1858)  *“(…)****during millions of generations individuals of a species will be occasionally born with some slight variation, profitable to some part of their economy”*** (Darwin and Wallace, 1858) |
| **KC2:** Individual (phenotypic) variation | *“(…)* ***those individuals with the lightest forms, longest limbs, and best eyesight, let the difference be ever so small****,* ***would be slightly favoured****, and would tend to live longer, and to survive during that time of the year* ***when food was scarcest****; they would also rear more young, which would tend to inherit these slight peculiarities”* (Darwin and Wallace, 1858) |
| **KC3:** Differential fitness (likelihood to survive and reproduce) | *“(…) those individuals with the lightest forms, longest limbs, and best eyesight, let the difference be ever so small, would be slightly favoured, and* ***would tend to live longer, and to survive during that time of the year when food was scarcest; they would also rear more young, which would tend to inherit these slight peculiarities”*** (Darwin and Wallace, 1858) |
| **KC4:** Heritable traits | *“(…) those individuals with the lightest forms, longest limbs, and best eyesight, let the difference be ever so small, (…) they would also rear more young,* ***which would tend to inherit these slight peculiarities”*** (Darwin and Wallace, 1858) |
| **KC5:**  Reproduction | *“Suppose in a certain spot there are eight pairs of birds, and that only four pairs of them annually (including double hatches) rear only four young, and that these go on rearing their young at the same rate, then at the end of seven years (a short life, excluding violent deaths, for any bird) there will be 2048 birds, instead of the original 16”* (Darwin and Wallace, 1858) |
| **KC6:** Selection pressure | *“But for animals without artificial means, the amount of food for each species must, on an average, be constant, whereas the increase of all organisms tends to be geometrical, and in a vast majority of cases at an enormous ratio”* (Darwin and Wallace, 1858) |
| **KC7:** Differential survival | *“(…)during millions of generations individuals of a species will be occasionally born with some slight variation, profitable to some part of their economy.* ***Such individuals will have a better chance of surviving****, and of propagating their new and slightly different structure (…)”* (Darwin and Wallace, 1858) |
| **KC8:** Differential reproduction | *“(…) those individuals with the lightest forms, longest limbs, and best eyesight, let the difference be ever so small, would be slightly favoured, and would tend to live longer, and to survive during that time of the year when food was scarcest;* ***they would also rear more young,*** *which would tend to inherit these slight peculiarities”* (Darwin and Wallace, 1858) |
| **KC9:** Frequency change | *“(…) during millions of generations individuals of a species will be occasionally born with some slight variation, profitable to some part of their economy. Such individuals will have a better chance of surviving, and of propagating their new and slightly different structure, and* ***the modification may be slowly increased by the accumulative action of natural selection to any profitable extent. The variety thus formed will either coexist with or, more commonly, will exterminate its parent form”*** (Darwin and Wallace, 1858) |
| **KC10:** Speciation | *“But at that time, I overlooked one problem of great importance (…). This problem is the tendency in organic beings descended from the same stock to diverge in character as they become modified. (…) The solution, I believe, is that the modified offspring of all dominant and increasing forms tend to become adapted to many and highly diversified places in the economy of nature”* (Barlow, 1958) |

**TABLE A2** Definition of each rubric item, its score contributing to the level of evolution understanding and examples based on students’ answers.

|  | **Criteria** | **Definition** | **Score** | **Examples** |
| --- | --- | --- | --- | --- |
| Predictions* | *Fittest* | Student writes and/or draws that the fittest phenotype will become the most frequent | 1 | Student P, SA target, post-test (LUENS=3): ***I expect to find more butterflies with big noses*** *since there are more flowers with long calyxes and the others will die*  Student A, SA control, pre-test (LUENS=2): *In 100 years,* ***I expect to find more butterflies with long proboscides*** *since there are more butterflies with short proboscides now, which will use more nectar from the flowers with short calyxes, leaving the butterflies with short proboscides without food, unlike those with long proboscides that will continue to feed themselves as before*  Student F, SA target, post-test (LUENS=5): ***I think I will find more butterflies with long proboscides*** *since this butterfly has a lot of food and, for this reason, will have offspring faster and these offspring will still have the initial food. And one year after that, more flowers will grow and these butterflies can get food. The other butterflies will be left with no food and only some of them will be able to survive*  Student C, SB target post-test (LUENS=4): *In 100 years, I expect to find butterflies with short proboscides since* ***there will be more flowers with short calyxes*** *and therefore these butterflies have food.* ***Since there are more butterflies with short proboscides, they help flowers with short calyxes to reproduce more*** |
|  | *Equilibrium* | Student writes and/or draws that the two phenotypes will become equally frequent | 0 | Student B, SA control, post-test (LUENS=0): ***I expect to find the same quantity of butterflies with long and short proboscides*** *since each butterfly can lay four eggs* |
|  | *Fixist* | Student writes and/or draws that the initially most frequent haplotype will continue to be the most frequent one | 0 | Student L, SA control, post-test (LUENS=0)*: I expect to* ***find 16 butterflies with short proboscides and 4 with long proboscides since each butterfly lays 4 eggs***  Student X, SB target, pre-test (LUENS=0)*:* *In one hundred years, there will be butterflies since butterflies live one year and then die, and in one hundred years* ***there will be the same number as today but four times more. I will find 16 with short beaks and 4 with long*** |
| Justifications | *Developmental* | Student states that the size of the proboscides depends on the individuals’ developmental stage | 0 | Student K, SB control, post-test (LUENS=0)*:* *There will be more butterflies with short beaks.* ***Because these are younger.*** *Also,* ***a butterfly with a long beak is older*** |
|  | *Teleological* | Student justifies her/his prediction with a purpose, need or goal | 0 | Student Y, SB target, post-test (LUENS=0): *I expect to find butterflies since these are living beings and* ***they need to stay alive. And more with short proboscides***  Student M, SB target, post-test (LUENS=0): *I think I am going to find those with short and long proboscides* ***since, like this, there is lots of biodiversity*** |
|  | *Resource availability* | Prediction is justified by resource availability | 1 | Student P, SA target, post-test (LUENS=3): *I expect to find more butterflies with big noses* ***since there are more flowers with long calyxes*** *and the others will die*  Student A, SA control, pre-test (LUENS=2): *In one hundred years, I expect to find more butterflies with long proboscides* ***since there are more butterflies with short proboscides now, which will use more nectar from the flowers with short calyxes, leaving the butterflies with short proboscides without food, unlike those with long proboscides that will continue to feed themselves as before***  Student F, SA target, post-test (LUENS=5): *I expect to find more butterflies with long proboscides since* ***this butterfly has a lot of food*** *and, for this reason, will have offspring faster, and these offspring will still have the initial food. And one year after that, more* ***flowers will grow and these butterflies can get food. The other butterflies will be left with no food*** *and only some of them will be able to survive*  Student C, SB target post-test (LUENS=4): *In one hundred years, I expect to find butterflies with short proboscides since* ***there will be more flowers with short calyxes*** *and therefore these butterflies have food.* ***Since there are more butterflies with short proboscides, they help flowers with short calyxes to reproduce more*** |
|  | *Differential survival* | Student mentions that individuals with the fittest phenotype will survive more, or those with the least fit phenotype will die more | 1 | Student P, SA target, post-test (LUENS=3): *I expect to find more butterflies with big noses since there are more flowers with long calyxes* ***and the others will die***  Student F, SA target, post-test (LUENS=5): *I expect to find more butterflies with long proboscides since this butterfly has a lot of food and, for this reason, will have offspring faster and their offspring will still have the initial food. And one year after that, more flowers will grow and these butterflies can get food. The other butterflies will be left with no food and* ***only some of them will be able to survive*** |
|  | *Differential reproduction* | Student mentions that individuals with the fittest phenotype reproduce more or have more offspring than those with the least fit phenotype | 2 | Student D, SB target, post-test (LUENS=4): *I expect to find those with longer proboscides in one hundred years since* ***these can produce more eggs.*** *As such,* *there will be more butterflies on the island. This is going to happen because those with the longest proboscides are more capable, I mean, they have more food.*  Student F, SA target, post-test (LUENS=5): *I expect to find more butterflies with long proboscides since this butterfly has a lot of food and, for this reason,* ***will have offspring faster*** *and their offspring will still have the initial food. And one year after that, more flowers will grow and these butterflies can get food. The other butterflies will be left with no food and only some of them will be able to survive*  Student C, SB target post-test (LUENS=4): *In one hundred years, I expect to find butterflies with short proboscides since* ***there will be more flowers with short calyxes*** *and therefore these butterflies have food.* ***Since there are more butterflies with short proboscides, they help flowers with short calyxes to reproduce more*** |

*Note*: The fittest haplotype was considered to be the one with the longest proboscides, except when other phenotypes were considered by the students and correctly justified with differential survival and/or reproduction. Boldface font indicates the sections from the students’ answers assigned to the rubric item.

Abbreviations: *, whenever drawn and written predictions differed, we considered the latter prediction to be the valid one; LUENS, level of evolution understanding; SA, School A; SB, School B; Target, classes subjected to the proposed educational activity; Control, classes not subjected to the proposed educational activity.

**Figure A3 -**Examples of answers given by students. (a) example of an answer with a *fixist* prediction (Student L, post-test, text translation: *I expect to find 16 butterflies with short proboscides and 4 with long proboscides since each butterfly lays 4 eggs*; LUENS= 0); (b) example of an answer with an *equilibrium* prediction and a *teleological* justification (Student M, post-test, text translation: *I think I am going to find those with short and long proboscides because, like this, there is lots of biodiversity*; LUENS= 0); (c) example of a *fittest* prediction justified by *resource availability*, *differential survival* and *differential reproduction* (Student F, post-test, text translation: *I expect to find more butterflies with long proboscides since this butterfly has a lot of food and, for this reason, will have offspring faster* *and their progeny will still have the initial food. And one year after that, more flowers will grow and these butterflies can get food. The other butterflies will be left with no food, and only some of them will be able to survive.* LUENS= 5); (d) example of a *fittest* prediction justified by *resource availability* and *differential survival* (Student P, post-test, text translation: *I expect to find more butterflies with big noses since there are more flowers with a long calyx and the others will die*; LUENS= 3).


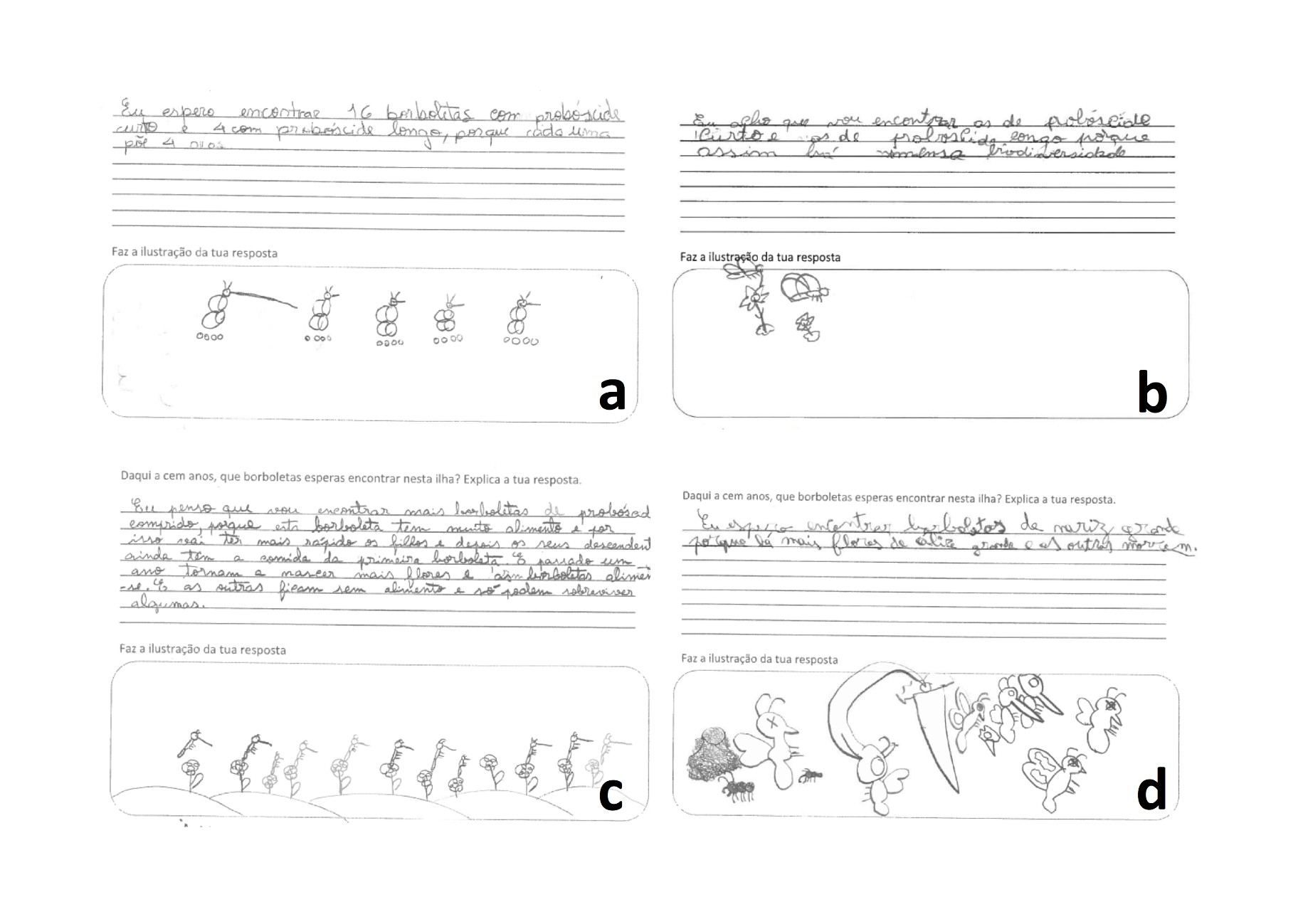


**Figure A4-** Evidence collected from class observations and students’ produced materials in the first session. In students’ materials, English translations of what was written by students are provided in the printwritting (a) example of a students’ pre-concept of a mite; (b) mite observed under a tripod magnifier; (c) students observing mites under a microscope; (d) preparation of mites to be observed with a magnifying camera; (e) example of how a group of students proposed to estimate mites’ size.


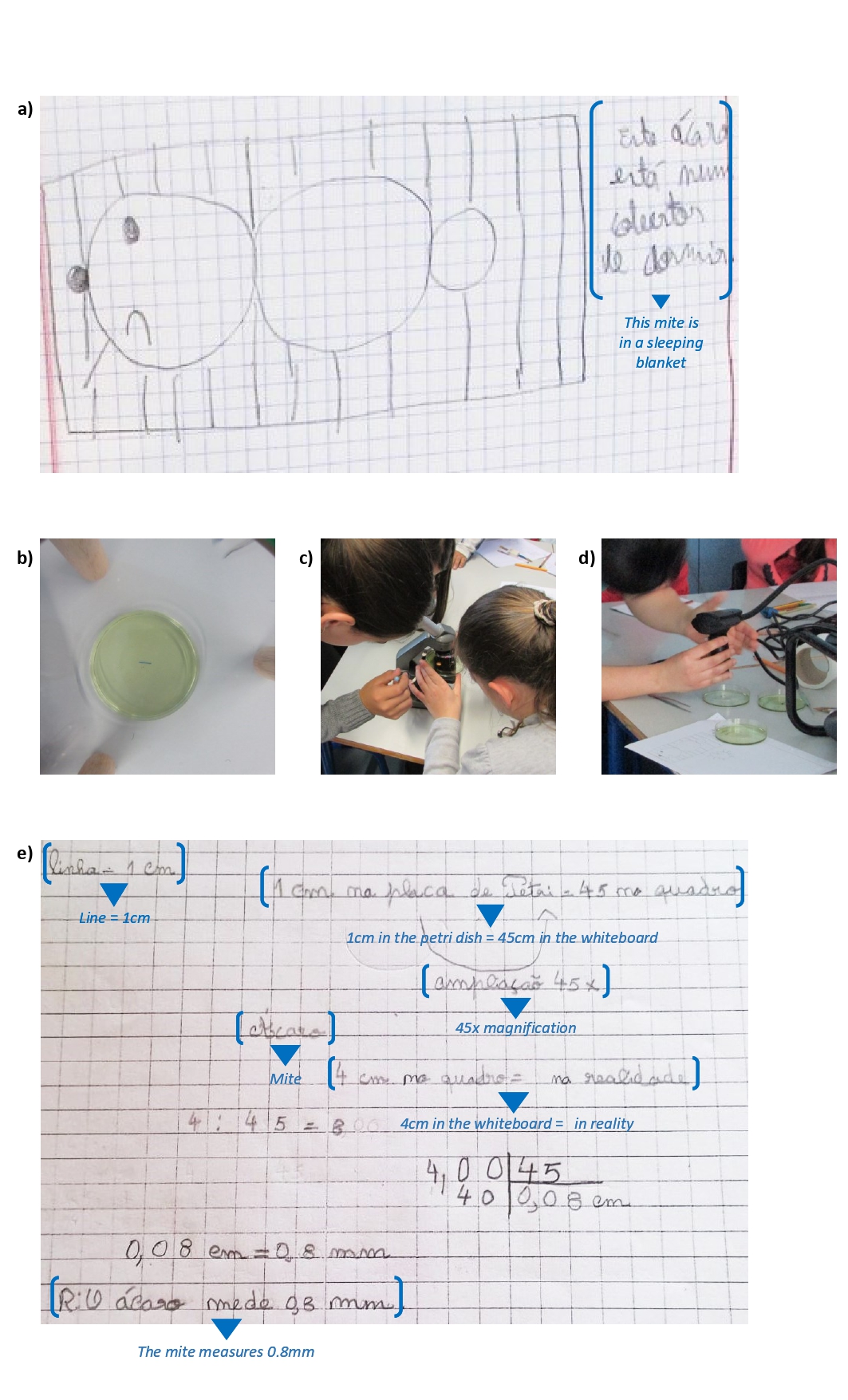


**FIGURE A5 -** Evidence collected from class observations and students’ produced materials in the second session. In students’ materials, English translations of what was written by students are provided in the printwritting (a) a group of students explaining how they estimated the mites’ population size using only mathematical language to their peers; (b) another group of students explain the strategy they used to estimate population size, using a combination of tables and mathematical language; (c) example of a students’ group work depicting the estimation and graphic representation of both mites population sizes.


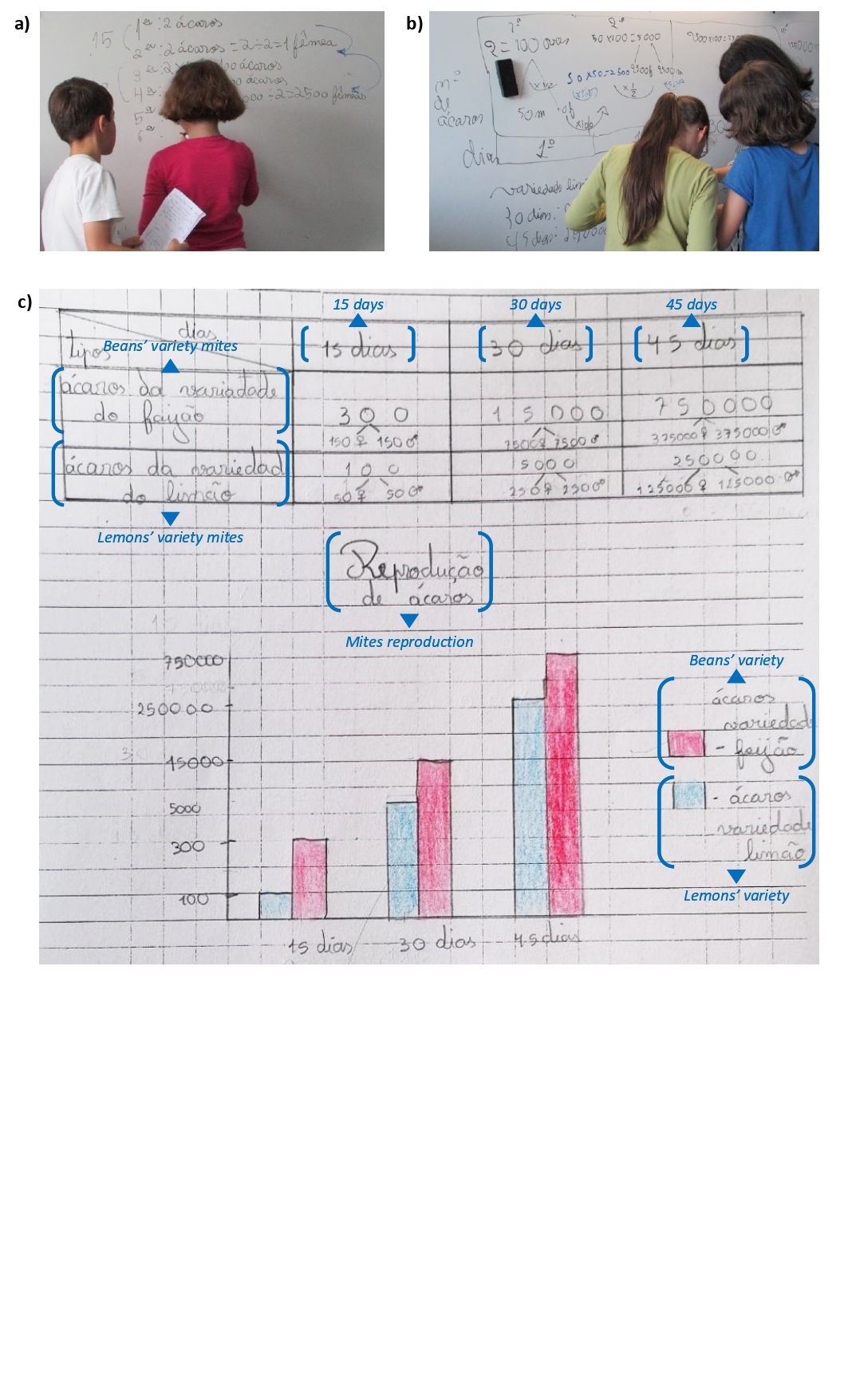


**FIGURE A6** Evidence collected from class observations and students’ produced materials in the third session. In students’ materials, English translations of what was written by students are provided in the printwritting (a) students’ estimated the space occupied by lemon and beans’ mites; (b) example of the work of one group of students, estimating the number of lemon and beans’ mites that could be supported by the available resources; (c) example of a students’ group work depicting the estimation of the population sizes of the two mites under resources limitation.


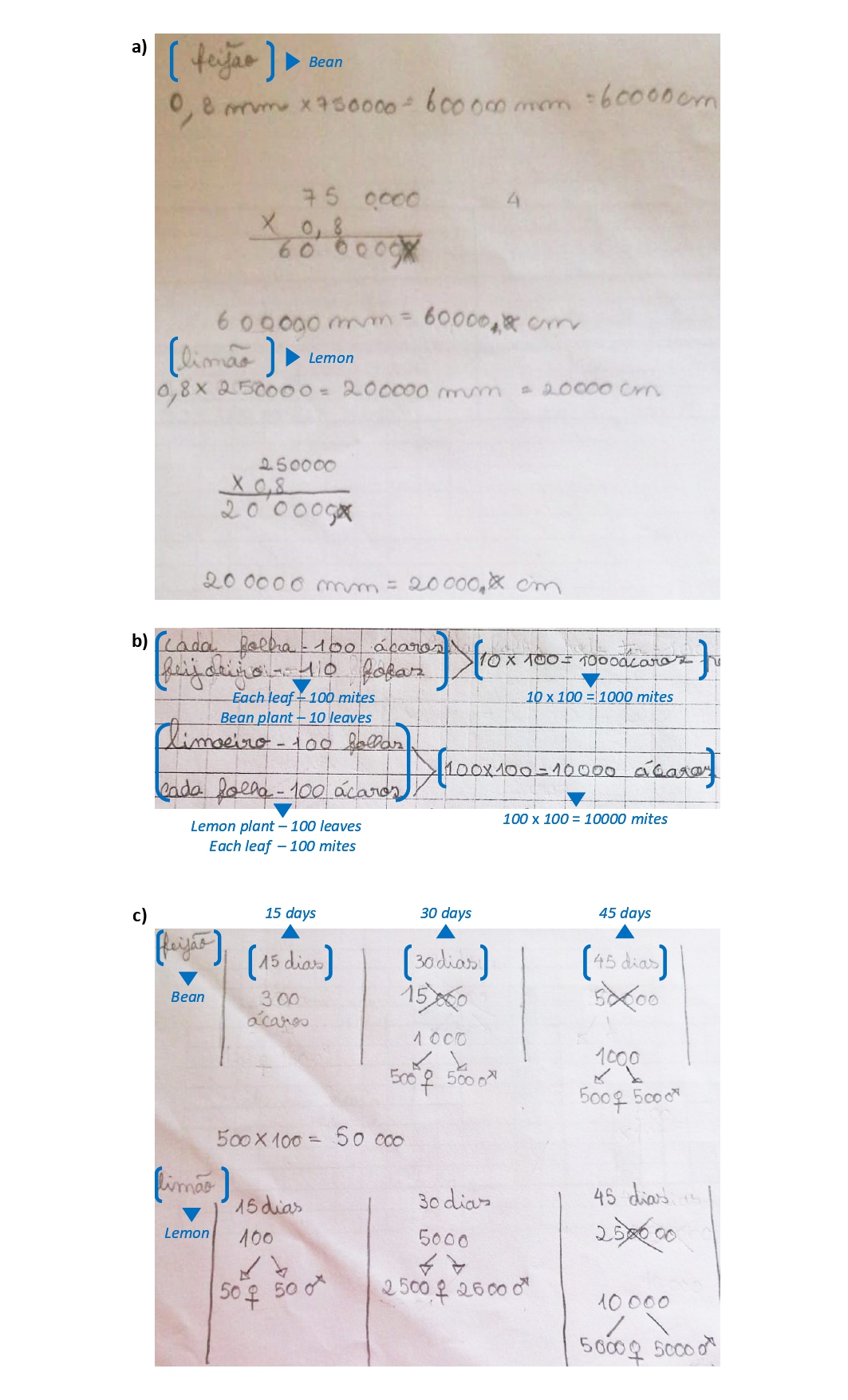


**TABLE A3** Results obtained for each class in pre- and post-tests for each rubric item, and p-values obtained in MacNemar tests

comparing the frequency of answers assigned to each coding rubric item between pre and post tests for each class (in brackets).

| **Class/**  **test** |  | **Fittest** | **Equilibrium** | **Fixist** | **Developmental** | **Teleological** | **Resource availability** | **Differential survival** | **Differential reproduction** |
| --- | --- | --- | --- | --- | --- | --- | --- | --- | --- |
| SAT  pre-test | N | 19 | 19 | 19 | 19 | 19 | 19 | 19 | 19 |
|  | % | 15.8 | 5.3 | 63.4 | 0 | 0 | 15.8 | 10.5 | 0 |
|  | IR | 1 | 1 | 1 | 1 | 1 | 1 | 0.95 | 0.95 |
| SAT  post-test | N | 19 | 19 | 19 | 19 | 19 | 18 | 19 | 19 |
|  | % | 68.4**  (0.006) | 0  (1.000) | 21.1*  (0.021) | 0  (#) | 0  (#) | 72.2**  (0.002) | 42.1*  (0.031) | 26.3  (0.063) |
|  | IR | 1 | 0.95 | 1 | 1 | 1 | 1 | 0.89 | 1 |
| SBT  pre-test | N | 24 | 20 | 23 | 25 | 21 | 25 | 25 | 24 |
|  | % | 41.7 | 0 | 34.8 | 0 | 0 | 36.0 | 8.0 | 8.3 |
|  | IR | 1 | 1 | 1 | 1 | 1 | 1 | 0.96 | 1 |
| SBT  post-test | N | 23 | 21 | 23 | 24 | 21 | 23 | 23 | 22 |
|  | % | 69.6*  (0.016) | 0  (#) | 13.0*  (0.031) | 4.2  (1.000) | 4.8  (1.000) | 60.9  (0.07) | 30.4  (0.125) | 27.3  (0.219) |
|  | IR | 0.96 | 1 | 1 | 1 | 1 | 0.96 | 0.96 | 0.95 |

Abbreviations: N, number of answers that could be classified for the rubric item; %, percentage of answers assigned to the rubric item; IR, interrater reliability; *, value significantly different from pre-test result with McNemar test p-values lower than 0.05; **, value significantly different from pre-test result with McNemar test p-values lower than 0.01. #, value that can not be estimated.

***Mathematical modelling and linkage to historically important key concepts***

During the first session, students from both classes observed mites using several magnifying devices (e.g., a magnifying glass, tripod magnifier, digital stereo microscope, and magnifying camera; see Figure A4 b,c,d). From the magnified image projected on the board, students successfully estimated the size of mites by applying two solutions to the same problem. In both cases, they used an object that was measurable in both the magnified and non-magnified images as a size scale. In SBT, they used a line drawn in the Petri dish by the researchers before the activity for this purpose. Although this line was drawn in all Petri dishes used in both classes, the students in SAT first proposed and used a piece of vegetable material in the petri dish. After finding a solution to determine the spatial scale of magnification, students mathematically estimated the size of the mite (Figure A4e). This allowed students to explore spatial scales and compare their previous ideas (Figure A4a) with real-life observations and their mathematical estimations.

In session 2, students estimated the number of mites of each variety that would be available for other students to observe in 45 days. When asked to consider the biological information they would need to make these estimations, students mentioned variables such as the number of progeny a mite could have, their life expectancy, the number of males and females in each population, the time it takes for the eggs to hatch, the age at which individuals start to reproduce (i.e., generation time) and the size of individuals. During the discussion, students decided that individuals’ size and hatching time were not necessary to solve the problem. Different groups of students presented various strategies to solve the problem and organise the data to their class, including strategies solely based only on mathematical language and strategies combining this with tables (Figure A5a and b, respectively). After identifying and discussing the correct approaches in both classes, the combination of mathematical language with tables was chosen by the students to perform the estimations for the second mite population (KC2, Table 1), reproduction (KC5, assuming the trait heritability KC4, Table 1) and population growth (Figure A5c).

In the third session, when asked about the type of biological information they would require to estimate the number of mites of each variety by the end of 45 days, students mentioned two additional parameters: the number of mites that could be accommodated on one leaf and the number of mites that one leaf could feed. In one of the classes (SAT), students initially estimated the space occupied by the mites estimated in the previous session (Figure A6a). However, after estimating the number of mites that could fit on one leaf, students noticed that leaf size would change since the mites feed on leaves. Accordingly, the students decided to instead use the number of mites that could be fed by one leaf to estimate the maximum number of each plant specialist mite that could survive based on the number of leaves supplied (Figure A6b). In the second class (SBT), the second approach was chosen by the students from the start. After estimating this parameter, students estimated the number of each mite variety after 45 days by applying a strategy similar to the one used in session 2 (again applying KC2, KC5 and KC4) while also accounting for resource availability (KC6, Table 1). When asked to explain why the least frequent variety of mites become the most frequent after this estimation (Figure A4c; KC9, Table 1), students introduced and discussed the concepts of differential survival (KC7, Table 1) and differential reproduction (KC8, Table 1) as well as their impacts on the number of offspring remaining over generations (KC3, Table 1). During this discussion, students in both classes orally described the process of natural selection applied to this biological scenario. These results suggest that during the three sessions, the students explored all of the key concepts that were planned (Table 1).
